# Special Issue: “Molecular Mechanisms of Plant Biostimulants”

**DOI:** 10.3390/ijms27010517

**Published:** 2026-01-04

**Authors:** Michael Moustakas, Julietta Moustaka

**Affiliations:** 1 Department of Botany, Aristotle University of Thessaloniki, 54124 Thessaloniki, Greece; moustak@bio.auth.gr; 2 Department of Food Science, Aarhus University, 8200 Aarhus, Denmark

Among the current methodologies proposed to increase plant resistance to abiotic stresses, the utilization of plant biostimulants in crop production stands out [1,2,3,4]. Plant biostimulants constitute an emerging class of agricultural inputs that aim to improve nutrient acquisition and increase crop quality and yield, while protecting the crops from abiotic stresses [5,6,7]. The main categories of biostimulants are protein hydrolysates, humic substances, seaweed and plant extracts, inorganic compounds, chitosan and biopolymers, and, lastly, microbial inoculants [8,9,10,11,12,13,14,15,16,17].

Today, biostimulants have become instrumental in the move towards sustainable agricultural approaches. Recent regulatory efforts, principally by the European Union with regulation 2019/1009, have helped in promoting their use in agriculture but uncertainties remain concerning their differentiation from fertilizers and plant protection products, and the evaluation of their effects is constrained by agronomic variability and the absence of standardized methodologies [18,19,20,21]. Moreover, the guiding background for biostimulants remains fragmented, which is a noteworthy obstacle to their universal acceptance [20]. Biostimulants, as mentioned above, encompass a broad range of products which complicates the efforts to establish standards that can homogenously be applied through all the types [22].

Today, there is a need to improve the current crop productivity to meet the increasing food demands [23,24,25]. The optimization of biostimulant utilization has great potential in an innovative and sustainable agriculture context, providing benefits to plant growth and health through increases in nutrient uptake, photosynthesis and secondary metabolism, conferring plant tolerance to environmental stresses [26,27,28,29].

To maximize the benefits of biostimulants, formulations must be tailored to the specific needs and growth circumstances of each crop species [20]. This level of customization necessitates a comprehensive understanding of the complex interactions between biostimulants and the distinct biochemical pathways of different crop species [20], thus elucidating their unique mode of actions. Achieving this understanding requires extensive research to systematically elucidate these interactions, thereby ensuring that biostimulant applications effectively and efficiently enhance plant health and productivity [30].

Confirmation from numerous studies reveals the benefits of biostimulants throughout crops [31]. Arbuscular mycorrhizal fungi (AMF) and plant growth-promoting rhizobacteria (PGPR) are emerging as novel approaches of biostimulants for improving crop quality and yield and also enhancing photosynthesis and environmental stress tolerance by activating genes responsible for antioxidant defense system [32,33,34,35]. Microbial biostimulants trigger plant growth through solubilization of minerals and boosting nutrient uptake, production of phytohormones, secondary metabolites, enzymes, volatile organic and inorganic compounds, carbohydrates, terpenoids, lipopolysaccharides, and photosynthetic capacity [34,36].

Low-temperature stress affects crop growth, development, yield, and quality of many crops [37], and, among others, the important oil crop, rapeseed. Zhu et al. [38] reported that the application of wood vinegar or butyrolactone (γ-Butyrolactone, one of the main components of wood vinegar) to rapeseed at the seedling stage promoted the growth and enhanced the low-temperature resistance by improving biomass, chlorophyll content, and soluble sugars, while also reducing membrane lipid oxidation. By using transcriptome and metabolomics analysis they revealed that wood vinegar improved growth through amino acid biosynthesis pathways and enhanced low-temperature tolerance via proline metabolism. Butyrolactone promoted growth through amino acid biosynthesis and improved low-temperature resistance by activating sphingolipid metabolism. The authors suggested the use of wood vinegar and butyrolactone as a new approach to increase rapeseed yield under low-temperature stress [38].

Metal-based nanoparticles (NPs) have shown to be suitable as fertilizers, enhancing crop productivity and reducing plant disease. It was proposed that they can be used as nanofertilizers, nanopesticides, or growth stimulators, since they pose a lesser risk of environmental contamination than the conventional agrochemicals [39,40] and, recently, new formulations based on nanotechnology are being explored for plant biostimulants [19,41]. In recent years, among the tested NPs, calcium hydroxide nanoparticles [Ca(OH)_2_ NPs] have attracted significant interest for their ability to impact plant photosynthesis and boost agricultural productivity [39]. Tryfon et al. [42] synthesized oleylamine-coated calcium hydroxide nanoparticles [Ca(OH)_2_@OAmNPs] and concluded that they can be used as photosynthetic biostimulants to enhance crop yield. The synthesized nontoxic Ca(OH)_2_@OAmNPs effectively regulated the non-photochemical quenching (NPQ) mechanism that resulted in a controlled increase in reactive oxygen species (ROS) generation that induced a hormetic response of photosystem II (PSII) function. A controlled increase in ROS triggers acclimation signaling for the regulation of a variety of physiological functions, including plant function and development [43]. The high anti-bacterial and anti-fungal efficacy of Ca(OH)_2_ NPs is mediated through a controlled increase in ROS [44]. Low levels of ROS exert beneficial action and are considered as hormetic molecules [45,46].

Iron (Fe), despite being the fourth-most abundant element in the Earth’s crust, has poor solubility under basic pH conditions commonly found in calcareous soils, making Fe deficiency a major agricultural issue worldwide [47]. Beneficial rhizosphere microorganisms (bacteria, fungi, and/or yeast) play a fundamental role in improving plant nutrition and in activating local and systemic responses to biotic and abiotic stresses [3,4,48,49,50,51]. Sevillano-Caño et al. [52] reported that the yeast *Debaryomyces hansenii* can function as a biofertilizer in iron-deficient soils by enhancing plant nutrition and stimulating growth. It achieves this by promoting root hair development, which are crucial for nutrient absorption, particularly under iron-deficient conditions. The activation of plant mechanisms to acquire more iron is achieved by increasing ferric reductase activity and the expression of iron-acquisition genes like *FRO1* and *IRT1*. Additionally, *D. hansenii* may also induce systemic resistance in plants and improve the overall crop production sustainability [52]. It is suggested that the use of *D. hansenii* as a biofertilizer offers a sustainable alternative to conventional chemical fertilizers, which can have negative environmental impacts [52].

The increased demands to feed a world population of 9.77 billion people by 2050 [53] will require more than 60% further increases in food production [54]. Noel et al. [54], by using radiocarbon flux analysis in soybean plants, show that wood vinegar (pyroligneous acid) enhanced plant growth by stimulating the biosynthesis of chlorophylls a, b, and β-carotene, which resulted to improved photosynthesis and biomass production and up 40% higher crop yield.

Salinization occurs in a wide range of climates being more prominent in arid and semi-arid regions [55]. In irrigated cropland owing to human activities, a “secondary” salinization becomes more severe, due to the progressive accumulation in the soil of toxic ions dissolved in the irrigation water [56]. This accumulation of soluble salts in the soil is one of the principal reasons for the low productivity of many essential crops [57]. Zuzunaga-Rosas et al. [56], in a review article, summarized the beneficial role of agroindustrial by-products (like seeds, husks, biomass and fruits) as sources for biostimulants, in boosting horticultural crops’ tolerance to abiotic stresses including salinity, by improving nutrient uptake, photosynthesis, and antioxidant defense, while regulating osmotic homeostasis. These waste-derived stimulants, especially protein hydrolysates, enhance growth, yield, and quality by activating stress-responsive genes, controlling ion transport, and osmoprotection, offering a sustainable solution to climate change impacts in agriculture [56].

This review elucidates the capacity of biostimulants to mitigate the adverse effects of biotic and abiotic stresses, improve nutrient use efficiency, and strengthen the resilience of agricultural systems. By critically examining recent advancements and proposing policy development, and field-level implementation, the review emphasizes the necessity of advancing biostimulant science to fully harness its potential in driving the transition toward sustainable agriculture.
